# Studying Differences in Pdu Microcompartment Expression in the Context of *pdu* Gene System Cloning/Transfer and PocR Activity

**DOI:** 10.1002/mbo3.70453

**Published:** 2026-11-10

**Authors:** Emma Kopp, Anh Nguyen, Ryan Allen, Daniel Edelman, Timothy Hunter, Anna Wilson, James W. Wilson

**Affiliations:** ^1^ Department of Biology, Mendel Hall Villanova University Villanova Pennsylvania USA

**Keywords:** FRT‐Capture, microcompartment, *pdu*, *pocR*, *Salmonella*

## Abstract

Metabolism of 1,2‐propanediol (1,2‐PD) in *Salmonella enterica* occurs via the activity of microcompartment (MCP) proteins encoded by the *pdu* gene system. In this report, we observed that *Salmonella* Typhimurium appears to display greater 1,2‐PD metabolism compared to *S*. Arizonae and *S*. Enteritidis. We were interested in whether species/strain differences in 1,2‐PD metabolism are also seen in respective *pdu* clones, as this could have implications for downstream studies and applications with these systems. We therefore cloned the 38‐kb *pdu/cob/cbi* regions from *S*. Arizonae, *S*. Enteritidis, and other *S*. Typhimurium strains as part of a first‐of‐kind comprehensive comparison. Remarkably, we found no differences in expression of the various cloned *pdu* systems. However, we observed that the *S*. Arizonae and *S*. Enteritidis clones appear to display a greater dependence on the Crp regulator as compared to the *S*. Typhimurium clone, and this dependence could be alleviated by overexpressing the PocR regulator. We found that lack of functional expression in *Pseudomonas putida* could be alleviated via PocR overexpression, demonstrating that the roadblock is at the transcriptional level. In *Pectobacterium carotovorum*, substitution of the native *pdu* promoter with a strong heterologous promoter allowed expression. A reporter assay system showed that *S*. Typhimurium PocR displayed higher transcriptional activation compared to *S*. Arizonae and *S*. Enteritidis PocR. Overall, this work demonstrates the central importance of the PocR regulator/transcriptional level of *pdu* expression and provides strategies to overcome *pdu* expression issues in other bacterial hosts.

## Graphical abstract

We observed significantly different levels of 1,2‐PD metabolism in *Salmonella* Arizonae and Salmonella Enteritidis compared to *Salmonella* Typhimurium. Based on this, we present a first‐of‐kind comprehensive comparison of cloned pdu/cob/cbi systems in a range of bacteria. We found that roadblocks to functional expression could be reversed via different approaches, including PocR overexpression and insertion of a heterologous constitutive promoter to express pdu genes. This work presents solutions to microcompartment expression barriers in bacteria and highlights the activity of PocR.

## 1 Introduction

Microcompartments (MCPs) are amembranous, protein‐based organelles used in bacteria for the compartmentalization of metabolic steps that produce toxic and/or volatile intermediates (Bobik et al. 2015; Kerfeld et al. 2018; Kennedy et al. 2021). Prominent examples include the carboxysome for carbon fixation, 1,2‐propanediol (1,2‐PD) utilization (*pdu*) MCPs, and ethanolamine utilization (*eut*) MCPs (Huffine et al. 2023; Stewart et al. 2020; Pokhrel et al. 2021). In the *Salmonella enterica* genome, the 23 genes encoding the *pdu* MCP system are located on a contiguous DNA segment that is immediately adjacent to the 20 *cob/cbi* genes that code for the synthesis of vitamin B12, an essential cofactor for 1,2‐PD metabolism via the *pdu* MCP (Chowdhury et al. 2014; Bobik et al. 1999; Cheng et al. 2011). The *pdu* system (and other MCP systems) can work as a cassette to be transferred across bacteria with functional expression in different bacterial backgrounds (Walter et al. 1997; Parsons et al. 2008; Graf et al. 2018). We previously cloned the *S. enterica* serovar Typhimurium *pdu/cob/cbi* system from the χ3477 (LT2‐derived) strain using the FRT‐Capture technique for in vivo cloning, and we transferred the system to a range of bacteria and observed MCP formation and associated 1,2‐PD metabolic activity across these species (Graf et al. 2018; Wilson 2021). This study demonstrated that this system could potentially be used in a wide range of gram‐negative cells for bacterial engineering purposes (though one species, *Pseudomonas putida*, did not display functional clone activity, suggesting possible limits/differences depending on the background) (Graf et al. 2018). Transcriptional expression of the *pdu* genes is activated by the PocR regulator protein, and the *pocR* gene is located within the *pdu* gene system (just upstream of the *pdu* operon and divergently transcribed) (Walter et al. 1997; Bobik et al. 1992; Chen et al. 1995, 1994). PocR is an AraC family transcriptional activator that binds 1,2‐PD via an N‐terminal domain, which in turn allows DNA binding (via a C‐terminal domain) at the *pdu* promoter to activate transcription and play its role as the major regulator of *pdu* gene expression (Walter et al. 1997; Bobik et al. 1992; Chen et al. 1995, 1994).

In this report, we observe that *S*. Typhimurium appears to display higher 1,2‐PD metabolism as compared to other *Salmonella* serovars, Arizonae and Enteritidis. This observation fueled our already significant interest in the cloning/transfer/comparison of a range of other *pdu* systems to test for differences in activity. Any differences in native *pdu* systems and their respective clones could have major implications for downstream studies and applications where a baseline level and/or a range of *pdu* expression could be desired, and evolutionary insights could also be affected by this as well (i.e., to what level is a given *pdu* region able to be expressed outside the species of genome origin in a range of other bacteria?). Therefore, we performed a first‐of‐kind comparison of multiple *pdu* clones in a range of bacteria, including a series of strains containing deletions of previously‐identified transcriptional regulators of the *pdu* system. We also present a method for inducible *pdu* gene expression using heterologous inducers and the application of this method in studying *pdu* expression roadblocks in different bacterial backgrounds (including *S*. Typhimurium Δ*crp*, *P. putida*, and *Pectobacterium carotovorum*). We respectfully note that this method provides differences compared to a previously reported system, where the method here uses different inducers and is designed for use across bacteria with cloned *pdu* regions (Kim et al. 2014). In addition, based on the original observation of *Salmonella* serovar variation in 1,2‐PD metabolism and given the central role of PocR in *pdu* genomic/clone activity, we constructed a system to test for differences in activation ability between different PocR proteins. This analysis suggests that there are differences in PocR activation ability among serovar genomes, and we discuss the ramifications of this in relation to *pdu* clones.

## 2 Materials and Methods

### 2.1 Strains, Plasmids, and Media

Bacterial strains and plasmids are listed in Table 1. Plasmids pBAD18 + *pocR* opt, pBAD18 + *pocR* sub, pJAK12 + *pocR* opt, and pJAK12 + *pocR* sub were constructed via commercial synthesis of the *S*. Typhimurium *pocR* gene fragment (*S*. Typhimurium genomic coordinates 2114399–2115324, with features noted below), initial cloning into plasmid pUC57‐Km, and subcloning into plasmid vector pBAD18 or pJAK12 at the KpnI sites in each (Genscript, Piscataway, NJ, USA). The *pocR* opt and *pocR* sub alleles contain optimal and suboptimal Shine–Dalgarno sequences, respectively, with the pocR sub allele also containing a GTG start codon (Figure A1). These alleles also contain a 6xHis epitope at the immediate C‐terminus. Plasmids pBAD18 + *pocR* ST nat, pBAD18 + *pocR* SA nat, and pBAD18 + *pocR* SE nat were constructed via commercial synthesis of the indicated serovar *pocR* genes but with the natural upstream regions (containing native translational signals) as indicated in Figure A2, and these alleles also contain the 6xHis epitope at the immediate C‐terminus. Deletion of *pdu* regulator genes from the *S*. Typhimurium chromosome was performed using standard recombineering techniques as described previously (Datsenko and Wanner 2000). Construction of the *pduC::lacZ* reporter was performed in the chromosome of *S*. Typhimurium strain LT2 (χ3447) via insertion of plasmid pCE36 as described previously (Ellermeier et al. 2002). Deletion of *pocR* (Δ*pocR*) from the reporter strain was performed using standard recombineering as described (Datsenko and Wanner 2000). LB medium was used for routine culture growth (10 g tryptone, 5 g yeast extract, and 5 g NaCl), and MacConkey plate medium containing 1,2‐PD as a carbon source was formulated as previously reported (Graf et al. 2018). The 1,2‐PD broth used for LacZ assays was formulated as follows: 1× M9 salts, 1 mM MgSO4, 0.5% succinate, 1% peptone, 500 nM vitamin B12, and 0.5% 1,2‐PD. Antibiotics were used at the following concentrations (micrograms per milliliter): ampicillin, 200; chloramphenicol, 10; kanamycin, 50; streptomycin, 125; and tetracycline, 15. Arabinose was used to induce *pocR* expression from the pBAD18 vector at 0.1% final concentration, and IPTG was used at 1 mM for *pocR* induction from pJAK12. Streak plate results were reproduced with multiple separate colony isolates (two to eight isolates) in independent streaking plate sets (two to four separate sets). These sets were streaked by at least two different members of the lab on separate occasions. In the figures here, a plate set is shown containing one to eight isolates from a given strain, as indicated. To quantify the result in Figure 1, we used Trigit software to calculate μH per pixel for each strain on the +B12 plate (using a box in equivalent uniform areas of each streak) (Tjandra et al. 2023). The *p* values were obtained from four separate streak plates using a two‐tailed *t*‐test.

**Table 1:** Strains and plasmids used in this study.

Strain	Reference
*S*. Typhimurium χ3477	Graf et al. (2018), an LT2‐derived strain
*S*. Typhimurium SL1344	Gulig and Curtiss (1987)
*S*. Typhimurium 14028	Jarvik et al. (2010)
*S*. Typhimurium JW2748	Rodriguez‐Rivera et al. (2014), strain R8‐0787
*S*. Typhimurium JW2759	DiMarzio et al. (2013), strain P0832868
*S*. Arizonae 13314	ATCC
*S*. Enteritidis 13076	ATCC
*S*. Typhimurium χ3477 Δ*pdu*	Graf et al. (2018)
S. Typhimurium χ3477 *pduC::lacZ* Δ*pocR*	This study
*S*. Arizonae Δ*pdu*	This study
*S*. Enteritidis Δ*pdu*	This study
*Escherichia coli* TOP10	Invitrogen/ThermoFisher, Inc.
*Salmonella bongori* 43975	ATCC
*Serratia marcescens* 14041	ATCC
Pseudomonas aeruginosa PAK	Graf et al. (2018)
*Pseudomonas fluorescens* 13525	ATCC
*Pseudomonas putida* 49128	ATCC
*Pectobacterium carotovorum* BP7003	ATCC
*S*. Typhimurium SL1344 Δ*pdu*	This study
*S*. Typhimurium SL1344 Δ*pdu* Δ*arcA*	This study
*S*. Typhimurium SL1344 Δ*pdu* Δ*fnr*	This study
*S*. Typhimurium SL1344 Δ*pdu* Δ*himD*	This study
*S*. Typhimurium SL1344 Δ*pdu* Δ*ptsN*	This study
*S*. Typhimurium SL1344 Δ*pdu* Δ*cpxR*	This study
*S*. Typhimurium SL1344 Δ*pdu* Δ*crp*	This study

Plasmid	Source/Reference
R995 (referred to as “R”)	Pansegrau et al. (1994)
R + *pdu* LT2	Termed R + *pdu* ST in Graf et al. (2018), *pdu* from χ3477 (LT2‐derived)
R + *pdu* LT2 Δ*pocR*	This study
R + *pdu* LT2 Cm‐pr::*pduA*	This study
R + *pdu* SL	This study, *pdu* from SL1344
R + *pdu* 14	This study, *pdu* from 14028
R + *pdu* 48	This study, *pdu* from JW2748
R + *pdu* 59	This study, *pdu* from JW2759
R + *pdu* SA	This study, *pdu* from S. Arizonae 13314
R + *pdu* SE	This study, *pdu* from S. Enteritidis 13076
pBAD18	Guzman et al. (1995)
pBAD18 + *pocR* opt	This study, C‐terminal 6xHis
pBAD18 + *pocR* sub	This study, C‐terminal 6xHis
pBAD18 + *pocR* ST nat	This study, C‐terminal 6xHis
pBAD18 + *pocR* SA nat	This study, C‐terminal 6xHis
pBAD18 + *pocR* SE nat	This study, C‐terminal 6xHis
pJAK12	Wilson et al. (1997)
pJAK12 + *pocR* opt	This study, C‐terminal 6xHis
pJAK12 + *pocR* sub	This study, C‐terminal 6xHis

**Figure 1:**
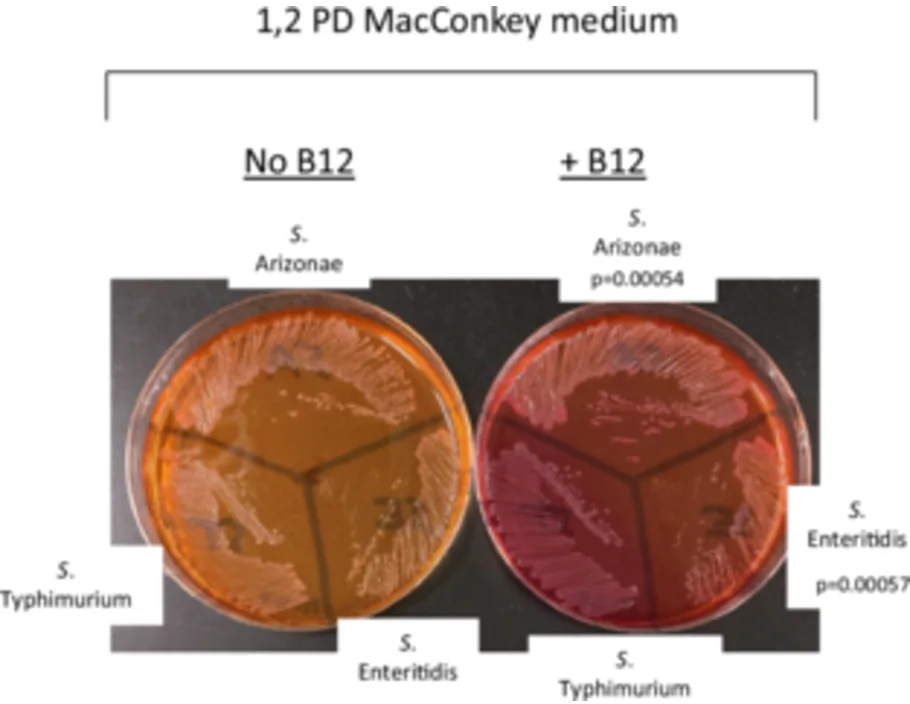
*Salmonella enterica* serovars Typhimurium, Arizonae, and Enteritidis were streaked onto 1,2‐PD MacConkey indicator medium either with or without vitamin B12. Metabolism of 1,2 PD is indicated by red color, and vitamin B12 is an essential cofactor for this pathway. To quantify the observed result, we used Trigit software to calculate μH per pixel for each strain on the +B12 plate (using a box in equivalent uniform areas of each streak) (Tjandra et al. 2023). The *p* values were obtained from four separate streak plates using a two‐tailed *t*‐test.

### 2.2 Cloning and Transfer of the *pdu/cob/cbi* Regions

The *pdu/cob/cbi* regions from *S*. Typhimurium strains, *S*. Arizonae, and *S*. Enteritidis were cloned into plasmid R995 (designated as “R”) using the FRT‐Capture technique as previously described (Graf et al. 2018). The strains *S*. Typhimurium Δ*pdu*, *S*. Arizonae Δ*pdu*, and *S*. Enteritidis Δ*pdu* were obtained as a by‐product of the FRT‐Capture procedure as previously described (Graf et al. 2018). An *E. coli* strain containing each clone was used as a donor in conjugative transfer to various gram‐negative recipients as described previously (Wilson et al. 2004; Wilson and Nickerson 2006a). All transconjugants were selected on M9 minimal medium (since the donor *E. coli* strain is auxotrophic) containing Tc and Km, except the conjugations to *S*. Typhimurium Δ*pdu*, *S*. Arizonae Δ*pdu*, and *S*. Enteritidis Δ*pdu*, which were selected on LB Cm Tc Km. Plasmid R + *pdu* LT2 Δ*pocR* was constructed using standard recombineering techniques (Datsenko and Wanner 2000). Plasmid R + *pdu* LT2 Cm‐pr::*pduA* was constructed using standard recombineering via a Cm‐R PCR product amplified from pKD3 such that it did not contain any FRT sites or possible transcriptional termination sequences so that downstream genes would be transcribed by the Cm‐R promoter (Datsenko and Wanner 2000). The Cm‐R product was inserted at homology sites between 56‐bp and 166‐bp upstream of the *pduA* gene (the first gene in the *pdu* operon).

### 2.3 MCP Preps, Protein Gels, and Western Blots

Isolation of intact MCPs from the indicated strains was performed using the MCP mini‐prep procedure as previously described (Graf et al. 2018). All cultures for MCP isolation were grown in LB medium either with or without 1% 1,2‐PD as indicated. Samples of MCP preps were run under standard denaturing conditions on an SDS‐PAGE gel and Coomassie stained as previously described (Graf et al. 2018). Western blots to detect PocR expression were performed using standard procedures as described previously (Solomon et al. 2014; Wilson and Nickerson 2006b; Wilson et al. 2007; Cangelosi et al. 2015) with total cell lysates from cultures grown in 1,2‐PD broth in the same strain background as for the LacZ assays. Quantification via ImageJ analysis was performed as previously described using the anti‐RpoS signal for normalization (Solomon et al. 2014; Schneider et al. 2012). Statistical analysis was performed using a standard two‐tailed *t*‐test of the indicated strains with six independent samples.

### 2.4 LacZ Assays

For LacZ assays, the indicated strains were grown overnight in 1,2‐PD broth (either with or without 1,2‐PD as indicated) from a fresh colony, and then this culture was diluted 1:50 and re‐grown to stationary phase (approximately 24 h) before an aliquot was used for LacZ assays as previously described (Kao et al. 2023). Statistical analysis was performed using a standard two‐tailed *t*‐test of the indicated strains with eight independent samples.

## 3 Results

### 3.1 Differences in *pdu* Expression Among *Salmonella* Serovars and Associated *pdu* Clones

In testing different strains for 1,2‐PD metabolism, we observed that *S*. Typhimurium appeared to display a greater amount of metabolism compared to *S*. Arizonae and *S*. Enteritidis on 1,2‐PD indicator medium (Figure 1) despite all three serovars containing the *pdu/cob/cbi* gene regions (Porwollik et al. 2002). On this indicator medium, a high level of 1,2‐PD metabolism will result in lower pH and an associated red color, whereas low/no level of this metabolism will result in peptone utilization, raised pH, and an associated white color (Parsons et al. 2008; Graf et al. 2018; Bobik et al. 1997). In addition, the absence of red color on the same indicator medium but with no added vitamin B12 confirms the B12‐dependent nature of the red phenotype, which is characteristic of 1,2‐PD metabolism (Graf et al. 2018; Bobik et al. 1992, 1997). We wondered if such differences were observed when the corresponding *pdu* regions were cloned and transferred to other bacteria, since this could have significant implications in downstream studies and applications of these clones. Therefore, we cloned the contiguous *pdu/cob/cbi* gene regions (38 kb segment) from the *S*. Arizonae and *S*. Enteritidis genomes using the in vivo cloning technique FRT‐Capture (Figure 2A). In addition, we also cloned the *pdu/cob/cbi* region from four other strains of *S*. Typhimurium, given that we have observed subtle differences among *S*. Typhimurium strains on indicator medium as well (our original clone was from a single strain, the LT2‐based χ3477) (Graf et al. 2018). The *S*. Typhimurium strains are the well‐known lab strains SL1344 and 14028, and the more recently isolated environmental/clinical isolates JW2748 and JW2759 (Gulig and Curtiss 1987; Jarvik et al. 2010; Rodriguez‐Rivera et al. 2014; DiMarzio et al. 2013). Strain JW2748 (referred to as strain R8‐0787 in (Rodriguez‐Rivera et al. 2014)) is a human clinical isolate, and strain JW2759 (referred to as strain P0832868 in (DiMarzio et al. 2013)) is an environmental isolate from cattle. The cloning inserts the *pdu/cob/cbi* region into the conjugative plasmid R995, and per the FRT‐Capture process, the initial host for this clone is the *pdu*‐negative species *Escherichia coli* (Graf et al. 2018). We tested for functional *pdu* expression in *E. coli* on MacConkey medium either with or without vitamin B12, a co‐factor essential for 1,2‐PD metabolism (Figure 2B). On this medium, the bacterial colonies/media turn a red color if 1,2‐PD is metabolized, whereas they display a white color if unable to perform this metabolism. Note that synthesis of vitamin B12 by the *cob/cbi* genes only occurs under anaerobic conditions (Jeter et al. 1984), and therefore under aerobic conditions (as used here), vitamin B12 needs to be supplemented in the medium for *pdu* system function to be detected. All the clones displayed a similar vitamin B12‐dependent red color on this medium (noting that the colonies on plates without B12 are white as expected and confirming that *pdu* is the cause of the red color) (Figure 2B).

**Figure 2:**
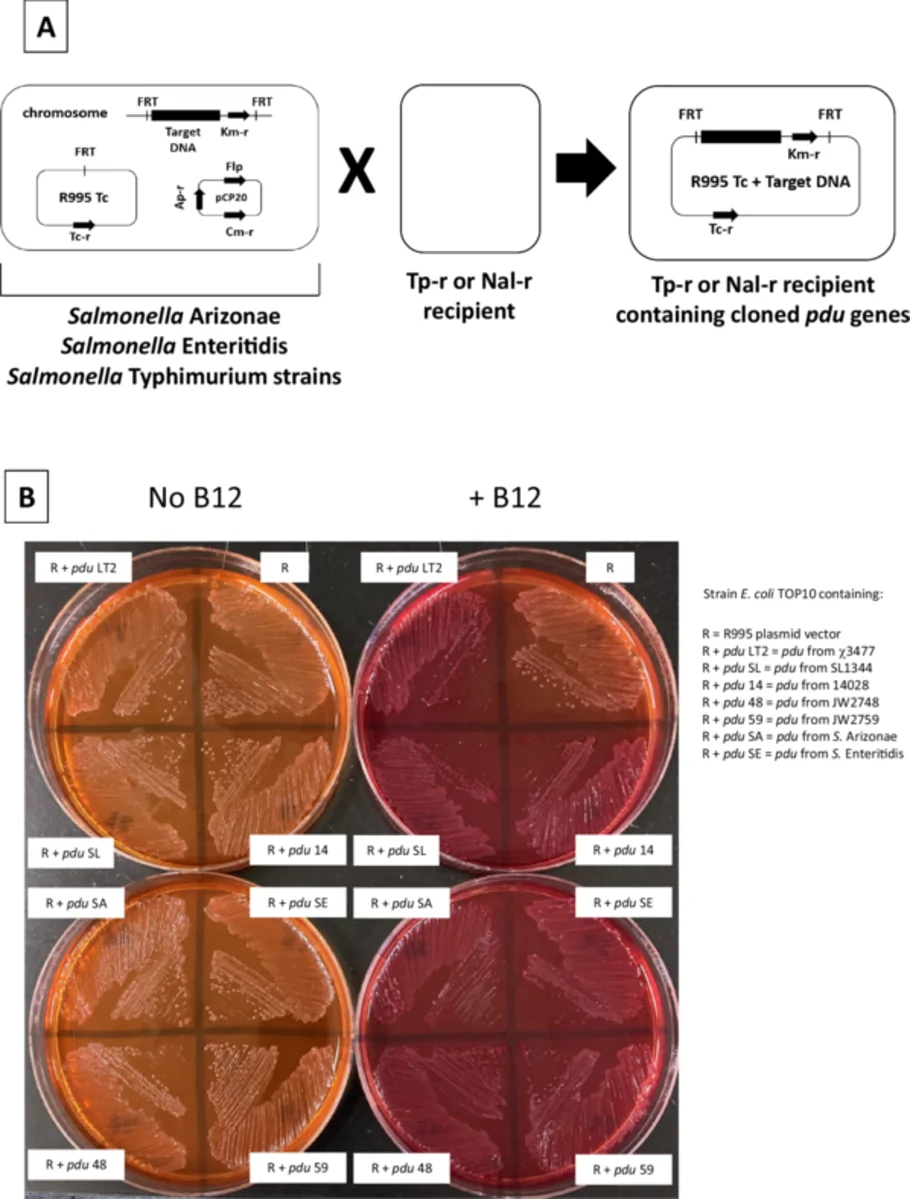
(A) The FRT‐Capture method for cloning large bacterial genomic fragments. FRT sites flanking target DNA (the *pdu/cob/cbi* region is 39 kb) are used to excise and insert the target DNA via Flp enzyme action into the plasmid vector R995. This construct is recovered via conjugation to a fresh *E. coli* Tp‐R or Nal‐R background. In this study, *S*. Arizonae, *S*. Enteritidis, and *S*. Typhimurium strains are the source strains for this technique. (B) The *E. coli* strain TOP10 (initial recipient strain) containing the indicated plasmids was streaked onto 1,2‐PD MacConkey medium either with or without vitamin B12. Red color indicates metabolism of the 1,2‐PD carbon source. The plasmid R refers to the plasmid vector R995, and the other plasmids are the *pdu* clones as indicated in Table 1 (and stipulated here in the label key).

To test if these clones displayed any differences in activity in different bacteria, we transferred these clones to a range of other gram‐negative strains/species and streaked on 1,2‐PD MacConkey plates (Figure 3). Except in *P. putida*, all the clones displayed a similar red color in the range of bacteria tested (Figure 3). In *P. putida*, all the clones displayed a white color, indicating a lack of *pdu* system activity, and this matches previous results in which the R + *pdu* LT2 clone displayed this phenotype (Figure 3) (Graf et al. 2018). It is remarkable that the expression issue in *P. putida* is seen with all the clones and indicates an expression, regulation, and/or enzymatic defect that occurs in this species (see below for further analysis).

**Figure 3:**
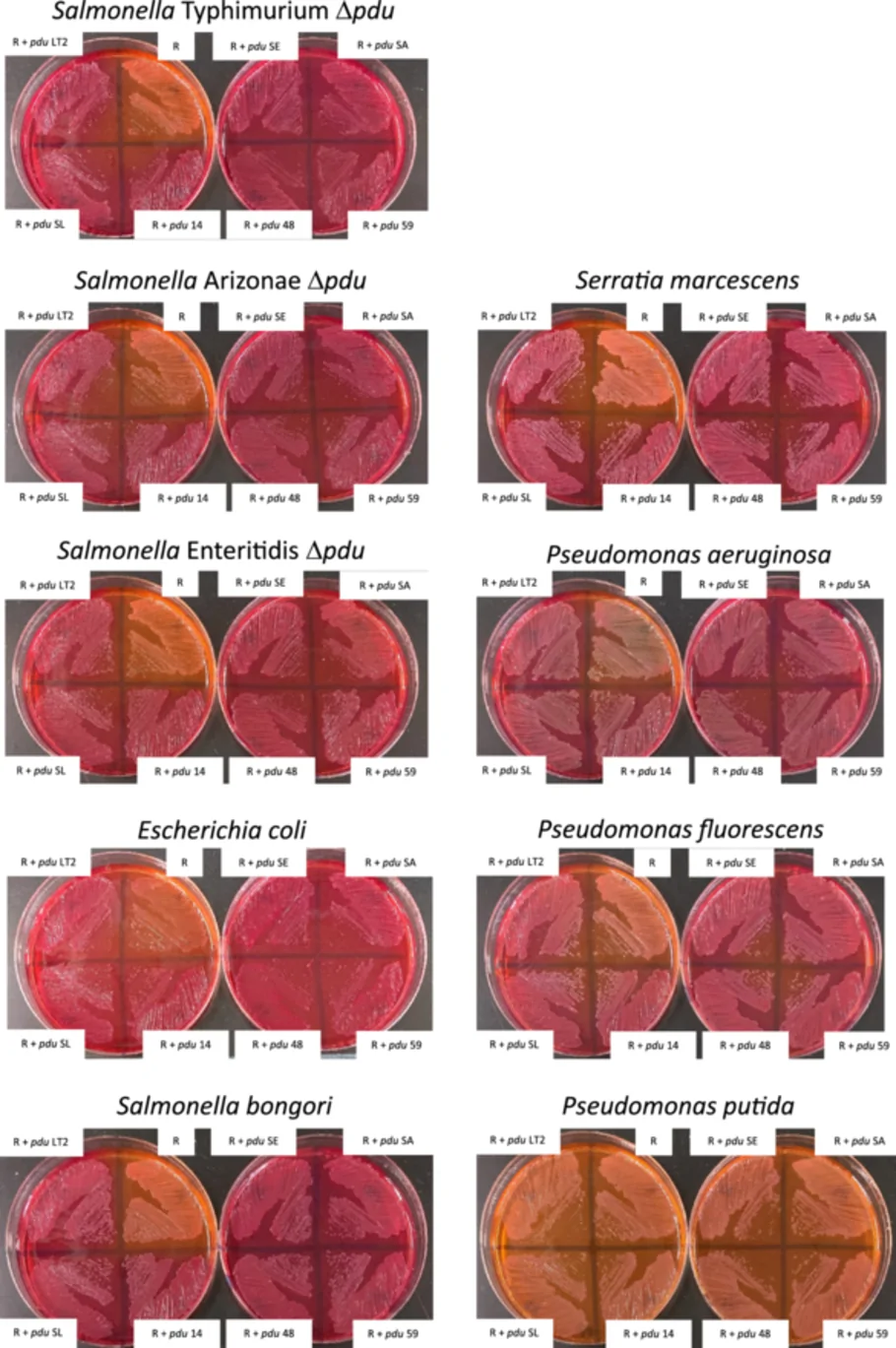
The indicated plasmids were each conjugatively transferred to the indicated strains, followed by colony purification and then streaked on 1,2‐PD MacConkey medium containing vitamin B12. The plasmid R refers to the plasmid vector R995, and the other plasmids are the *pdu* clones as indicated in Table 1 and Figure 1. Each strain was also tested on 1,2‐PD MacConkey medium without vitamin B12, and all displayed white color on this medium (Graf et al. 2018).

Even though we observed the same red color on indicator plates, it is possible that there could be differences in the levels of actual MCPs expressed/formed from the different clones. Therefore, we isolated MCPs expressed from each clone and compared them via SDS‐PAGE and Coomassie staining (Figure 4). We observed the previously‐identified Pdu MCP protein bands for each clone in this analysis (Figure 4). Interestingly, the approximately 17–18 kD band appears shifted up in size in the MCP prep from the *S*. Arizonae clone and likely corresponds to PduE, which is predicted to be a larger protein in *S*. Arizonae from the gene sequence. We do not know how MCP protein differences in the *S*. Arizonae clone (or possibly other clones) could translate to a difference in *pdu* system activity.

**Figure 4:**
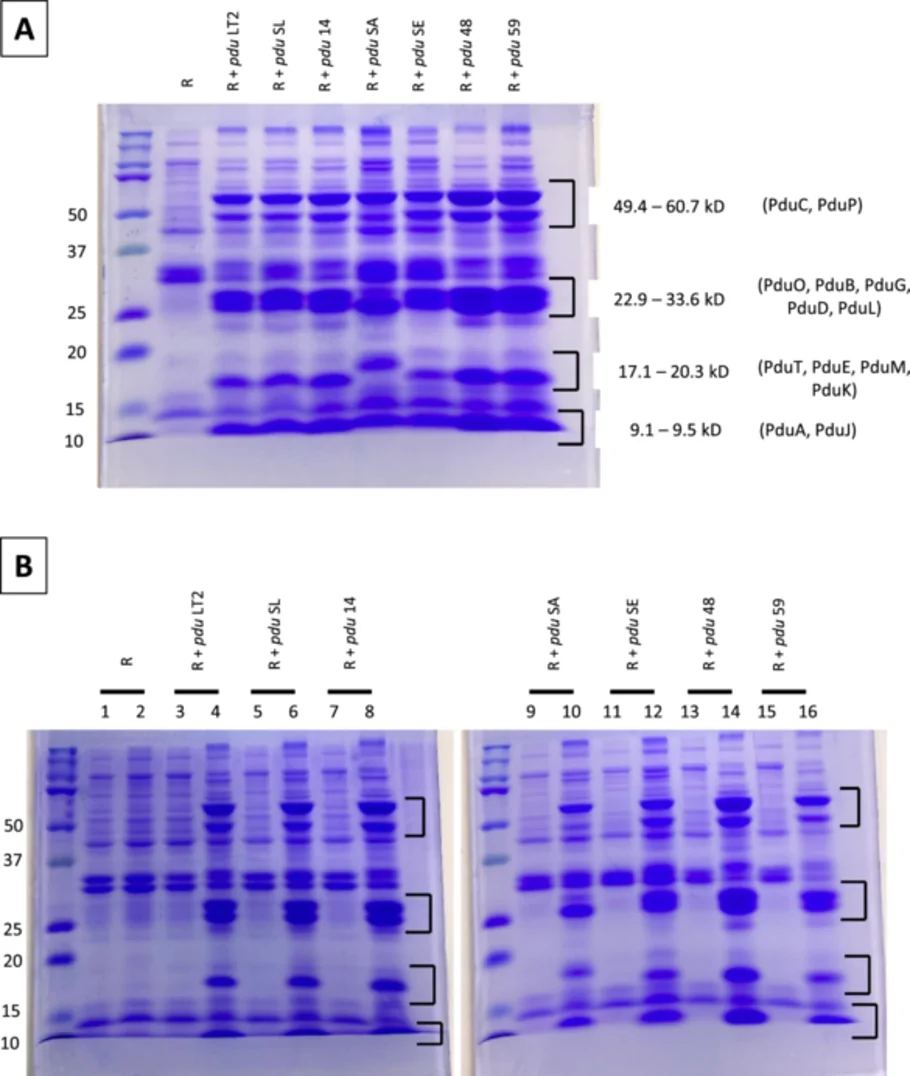
(A) MCP preps were performed with *E. coli* strain TOP10 containing the indicated plasmids grown in LB medium containing 1,2‐PD, and MCP samples were run via SDS‐PAGE and Coomassie‐stained. The plasmid R refers to the plasmid vector R995, and the other plasmids are the *pdu* clones as indicated in Table 1 and Figure 1. Size markers (in kilodaltons) are indicated on the left side of the gel, and the brackets on the right side of the gel indicate MCP protein bands identified via mass spectrometry, as in Graf et al. (2018). (B). MCP preps were performed with the strains indicated in (A) above, grown in LB medium either with or without 1,2‐PD. Samples were analyzed via SDS‐PAGE and Coomassie staining. Odd‐numbered lanes are cultures without 1,2‐PD, and even‐numbered lanes are cultures supplemented with 1,2‐PD. Size markers and brackets for MCP protein bands are indicated as in (A).

Expression of the chromosomal *pdu* system is induced by the presence of the 1,2‐PD molecule (and is off in the absence of 1,2‐PD) (Bobik et al. 1992). It is critical to test if such regulation is still intact when cloning large gene systems as we have previously observed pertubations to regulation schemes in similar types of clones (Wilson and Nickerson 2006a, 2006b; Wilson et al. 2007; Cangelosi et al. 2015; Patel et al. 2020). To test if this level of regulation is observed in the *pdu* clones, we grew strains in either the absence or presence of 1,2‐PD and performed MCP preps for each population (Figure 4B). We observed clear differences between the two conditions for each *pdu* clone that likely indicate lower or no *pdu* gene expression in the absence of 1,2‐PD and are consistent with the 1,2‐PD regulation paradigm being intact for each clone. We note that this observation is consistent with RT‐PCR confirmation of this phenomenon at the transcriptional level as previously shown for the R + *pdu* LT2 clone (Graf et al. 2018).

A number of DNA‐binding transcriptional regulators have been implicated in the regulation of *pdu* gene expression including PocR (encoded within the *pdu* region), Crp, IHF, ArcA, PtsN, FNR, and CpxR (Bobik et al. 1992; Chen et al. 1995; Ailion et al. 1993; Rondon and Escalante‐Semerena 1997; Wang et al. 2019; Subramaniam et al. 2019; Yoo et al. 2017). Since the cloning of the *pdu* region involves removal of the genes from their normal chromosomal context and maintenance at a higher copy number on plasmid R995 (approximately 10 copies/cell), it is relevant to test the roles of these individual regulators for cloned *pdu* expression. To test this, we constructed deletions in the genes coding for Crp, IHF, ArcA, PtsN, FNR, and CpxR and transferred them to the *S*. Typhimurium Δ*pdu* background, and then we transferred the R + *pdu* LT2, R + *pdu* SA, and R + *pdu* SE clones into these strains. For PocR, we constructed a deletion of the cognate gene in the R + *pdu* LT2 clone. Analysis of the R + *pdu* LT2 Δ*pocR* plasmid demonstrated loss of *pdu* activity and confirmed that the essential nature of PocR for *pdu* expression is still intact from R995 (Figure 5A). Deletion of *arcA, ptsN*, *fnr*, *cpxR*, and *himD* (subunit of IHF) did not affect expression of the *pdu* clones tested (Figure 5B). However, in the Δ*crp* strain, we observed loss of *pdu* activity for the *S*. Arizonae and *S*. Enteritidis clones but not for the *S*. Typhimurium clone (Figure 5C). This uncovers a greater dependence on Crp for the *S*. Arizonae and *S*. Enteritidis clones as compared to *S*. Typhimurium (see further study of this result below). The results also indicate that the roles of ArcA, PtsN, FNR, CpxR, and IHF (in aerobic conditions as tested here) are minimal/absent for the cloned *pdu* genes.

**Figure 5:**
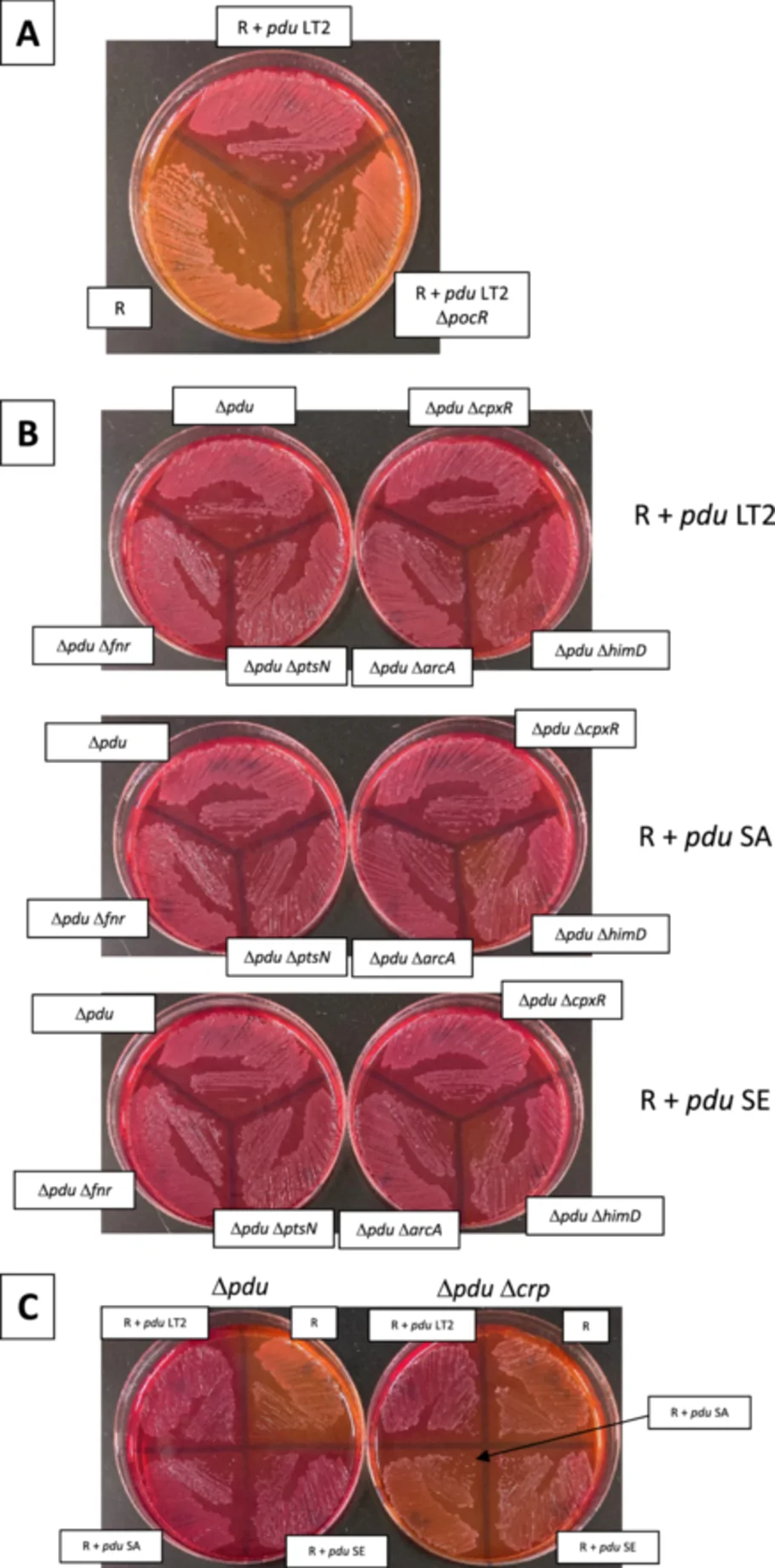
(A) The *E. coli* strain TOP10 containing the indicated plasmids was streaked onto 1,2‐PD MacConkey medium containing vitamin B12. (B) The *S*. Typhimurium Δ*pdu* strain containing the indicated mutations and harboring the indicated plasmids was streaked onto 1,2‐PD MacConkey medium containing vitamin B12. (C) The *S*. Typhimurium Δ*pdu* and Δ*pdu* Δ*crp* strains containing the indicated plasmids were streaked onto 1,2‐PD MacConkey medium containing vitamin B12.

### 3.2 Design of an Inducible *pdu* Expression System and Use With Δ*crp* Observation

The *pdu* genes are induced by the presence of the system's natural substrate 1,2‐PD (Bobik et al. 1992), and we show that this level of regulation is intact for the *pdu* clones in Figure 4 above. However, the engineered use of cloned *pdu* systems may necessitate heterologous induction of *pdu* gene expression so that the system can be off even in the presence of 1,2‐PD and then induced by a different molecule when needed. We designed such a system by cloning the *S*. Typhimurium *pocR* gene downstream of inducible promoters on the plasmids pBAD18 and pJAK12, which can induce gene expression via arabinose and IPTG, respectively. We respectfully note that a similar approach was previously used to induce the chromosomal *S*. Typhimurium *pdu* system (Kim et al. 2014); however, the design here utilizes different inducers and is for use with cloned *pdu* systems across bacteria (as used below in *E. coli*, *S*. Typhimurium, *P. putida*, and *P. carovortorum* backgrounds). Our initial *pocR* clone was engineered with an optimal Shine–Dalgarno sequence to maximize PocR translation (and the allele is denoted “*pocR* opt”). We demonstrated successful use of this construct by maintaining either pBAD18 + *pocR* opt or pJAK12 + *pocR* opt in cells with the R + *pdu* LT2 Δ*pocR* construct (Figure 6A). The cloned *pdu* system activity is restored by the *pocR* opt allele; however, we observed leaky Pdu activity in the absence of the inducer, though at a lower level (Figure 6A). To address this, we then designed a *pocR* allele (denoted “*pocR* sub”) with suboptimal translational signals (weak Shine–Dalgarno and GTG start codon), and we cloned this allele in an identical manner into both pBAD18 and pJAK12. When the *pocR* sub constructs were used in combination with R + *pdu* LT2 Δ*pocR*, Pdu activity was successfully induced in the presence of either arabinose or IPTG for each of these strains and was off in their absence (even though the natural inducer 1,2‐PD is present) (Figure 6A).

**Figure 6:**
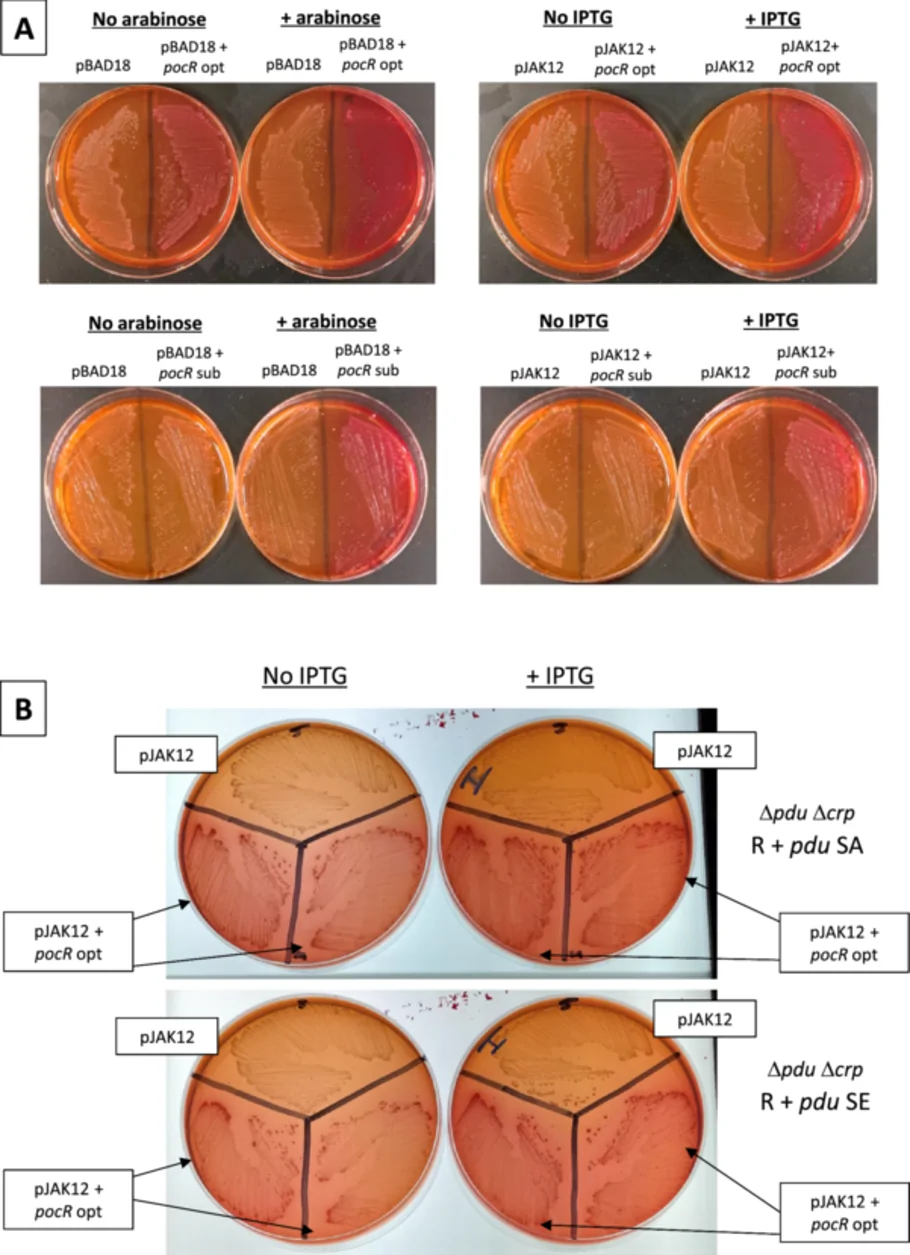
(A) The plasmid R + *pdu* LT2 Δ*pocR* was maintained with plasmid pBAD18 + *pocR* opt, pBAD + *pocR* sub, pJAK12 + *pocR* opt, or pJAK12 + *pocR* sub (or the corresponding vectors) in *E. coli* TOP10 and streaked onto 1,2‐PD MacConkey medium containing the indicated inducer (or not containing the inducer). (B) Strain *S*. Typhimurium Δ*pdu* Δ*crp* containing either R + *pdu* SA or R + *pdu* SE and either pJAK12 or pJAK12 + *pocR* opt as indicated was streaked onto 1,2‐PD MacConkey medium supplemented with vitamin B12 and either with or without IPTG. The pJAK12 + *pocR* opt streak sections are two separate isolates of the same strain.

To determine if the Δ*crp* phenotypes of R + *pdu* SA and R + *pdu* SE could be reversed by induction of the *pocR* gene via the system described above, we introduced these *pdu* clones into the *S*. Typhimurium Δ*pdu* Δ*crp* strain along with the pJAK12 + *pocR* opt plasmid. We note that, initially, we used the pJAK12 + *pocR* sub construct for this experiment, but this construct gave inconsistent results, likely due to the lower overall levels of PocR expression from the *pocR* sub allele (which appear to be unsuitable for this particular experiment). When streaked to indicator plates, we observed that pJAK12 + *pocR* opt restored the red color phenotype to R + *pdu* SA and R + *pdu* SE (Figure 6B). Note that we saw this result in both the presence and absence of IPTG, consistent with the leaky nature of pJAK12 + *pocR* opt (though there appears to be slightly more red on the plates with IPTG in Figure 6B). This result establishes a strategy that can be used to overcome barriers to cloned *pdu* expression when transferred to non‐expressing/lower‐expressing strains.

### 3.3 Analysis of pdu Expression Roadblocks in *P. putida* and *P. carotovorum*

Results in Figure 3 indicate that *P. putida* displays a lack of cloned pdu expression. This result is of significant interest since we can use this strain to study potential reasons for this phenomenon and develop strategies that would allow us to overcome it for subsequent bacterial engineering in this species (or any other non‐expressing species). Possible reasons for lack of cloned *pdu* expression in *P. putida* could be at the transcriptional level (promoter not working, RNA polymerase processing issues, etc.) and/or at the posttranscriptional level (mRNA made, but not stable or translated efficiently; proteins translated, but unstable or non‐functional; etc.). This distinction has implications for developing strategies to overcome the *pdu* expression issue since each level would utilize different approaches/experiments/techniques. One possible strategy would be to introduce a co‐resident PocR‐expressing plasmid, induce PocR expression, and test for 1,2‐PD metabolism on indicator medium. Therefore, we established R + *pdu* LT2 and pJAK12 + *pocR* opt (or pJAK12 vector as control) together in *P. putida* and tested this strain on 1,2‐PD indicator plates either with or without IPTG. We used the pJAK12 vector since it is more amenable to cross‐species use, and we used the *pocR* opt allele since this relatively distantly related species may need optimal signals to achieve PocR translation. The presence of pJAK12 + *pocR* opt resulted in the ability for *P. putida* to utilize 1,2‐PD, as seen on indicator medium with or without IPTG (indicating leaky *pocR* expression from pJAK12) (Figure 7A). This result indicates that the nature of the *pdu* expression roadblock in *P. putida* is at the transcriptional level and can be overcome via PocR overexpression.

**Figure 7:**
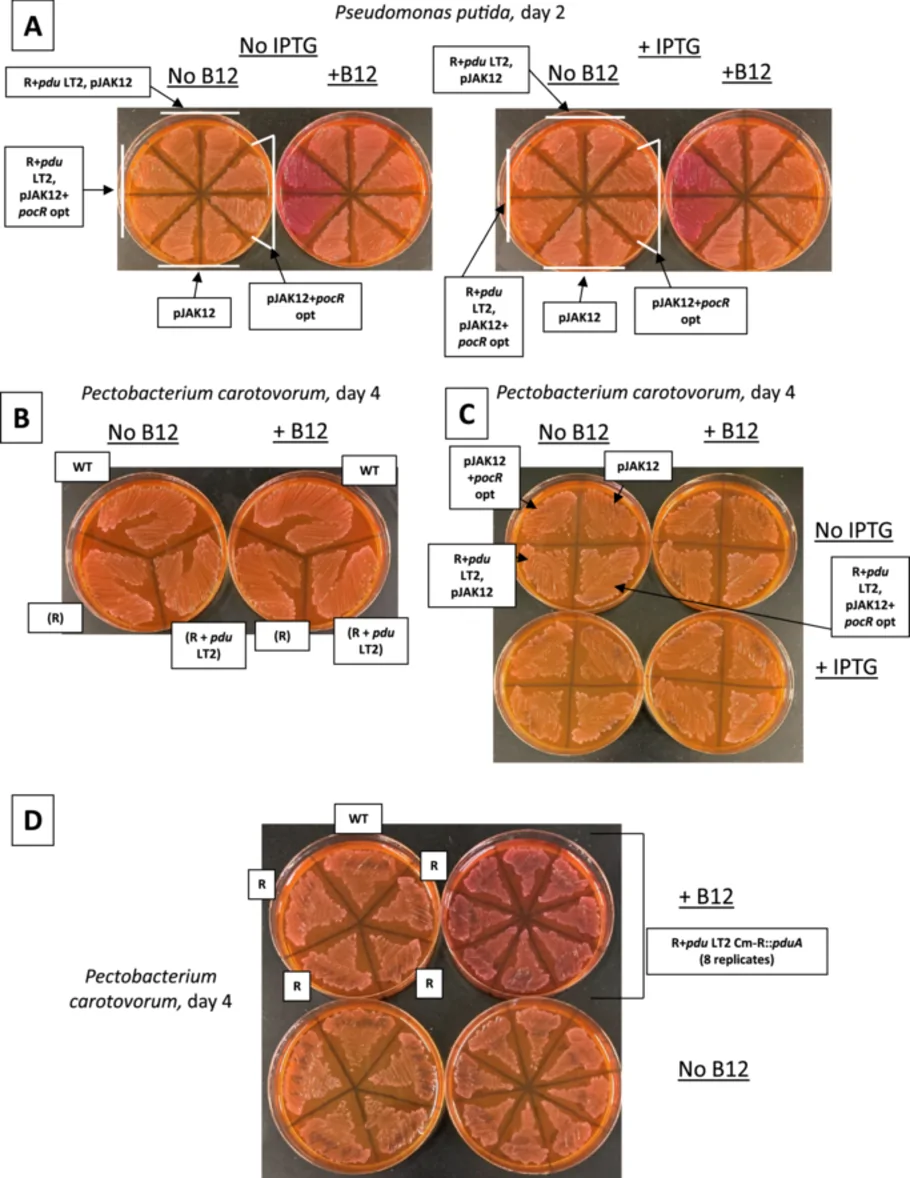
(A) *Pseudomonas putida* strains containing the indicated plasmids were streaked onto 1,2‐PD MacConkey medium with or without IPTG and with or without vitamin B12. Note that the labeling scheme on the left‐hand plate also applies to the right‐hand plate in the photos. The white lines indicate duplicate isolates of the indicated strain. (B) *Pectobacterium carotovorum* strains (either WT or containing the indicated plasmids) were streaked onto 1,2‐PD MacConkey medium either with or without vitamin B12. (C) *P. carotovorum* strains containing the indicated plasmids were streaked onto 1,2‐PD MacConkey medium with or without IPTG and with or without vitamin B12, as shown. The labeling scheme used in the upper left plate applies to the other plates as well. (D) *P*. *carotovorum* strains containing the indicated plasmids were streaked onto 1,2‐PD MacConkey medium either with or without vitamin B12. Note that the labeling for the upper plates applies to the lower plates as well and that there are 4 and 8 replicates of the “R” and “R + *pdu* LT2 Cm‐R::*pduA*” strains, respectively.

We then screened a series of other more distantly related (from *Salmonella*) bacterial species for 1,2‐PD metabolism on indicator plates. From this analysis, we discovered that *P. carotovorum* displayed no 1,2‐PD metabolism either endogenously in the WT strain or in strains containing cloned *pdu* (Figure 7C). This prompted us to introduce pJAK12 + *pocR* opt into this species to see if it could allow this phenotype to be overcome and allow coresident cloned *pdu* expression (as seen above with *P. putida*). Interestingly, we did not see this occur; induced PocR did not allow red color on the indicator medium in the cloned *pdu* strain (Figure 7D). To confirm that PocR protein is expressed in the appropriate *Pectobacterium* strains, we performed Western blots on total cell lysate samples from pJAK12 + *pocR* opt strains and relevant controls, and this analysis confirmed PocR expression (Figure A3). Thus, it appears that PocR overexpression is unable to restore 1,2‐PD metabolism in *Pectobacterium* strains containing cloned *pdu* and suggests that there are different types of transcriptional‐level roadblocks in non‐expressing species.

We then figured that if the PocR‐activated native *pdu* promoter cannot function in *Pectobacterium*, then maybe a heterologous, constitutive promoter could work in its place. We therefore constructed an R + *pdu* LT2 plasmid in which the native *pdu* operon promoter (just upstream of *pduA*) was replaced with the constitutive promoter that expresses the *cat* Cm‐R gene (from plasmid pKD3 as described in Section 2). Remarkably, this construct was able to achieve cloned *pdu* expression in *Pectobacterium,* as observed on indicator plates (Figure 7E). This result indicates another strategy for overcoming transcriptional‐level roadblocks to cloned *pdu* expression, where the PocR/*pdu* promoter circuit is replaced by another promoter system.

### 3.4 Are There Differences in PocR Activation Ability When Comparing *pocR* Genes From Different *Salmonella* Serovars?

Given the original observation in Figure 1, the differences in PocR/Crp dependence seen in Figures 5 and 6, and the different roles for PocR in cloned *pdu* expression in *P. putida* and *P. carotovorum* in Figure 7, we wondered if there was variation in activation ability in PocR proteins expressed from different serovar *pocR* genes. To test this, we constructed a reporter strain (in *S*. Typhimurium) that contained a chromosomal *pduC::lacZ* transcriptional fusion (*pduC* is the third gene in the *pdu* operon) and a chromosomal Δ*pocR* mutation (Figure 8A). Then, we could introduce each cloned *pdu* region from *S*. Typhimurium, *S*. Arizonae, and *S*. Enteritidis (which contain each respective *pocR* gene) and compare activation activity via the LacZ reporter. Using such a reporter scheme has the potential to reveal differences in *pdu* operon activation that may not be seen on indicator medium or via MCP preps, or it may provide further insight into any phenotypes seen via these techniques. After individual transfer of the cloned *pdu* regions from each serovar to the reporter strain (and vector‐only control), we assayed each strain for LacZ activity in 1,2‐PD broth either with or without 1,2‐PD. The results indicate greater *pdu* operon activation ability for the *S*. Typhimurium PocR compared to *S*. Arizonae and *S*. Enteritidis PocR (Figure 8B). This result is consistent with the Figure 1 observation and the greater dependence of *S*. Arizonae and *S*. Enteritidis cloned *pdu* on the Crp activator seen in Figure 5. One explanation for this result could be differences in PocR protein expression or stability. To test this, we performed Western blot analysis using *pocR* genes from each serovar expressed from their natural translational signals (expressed from plasmid pBAD18). This analysis revealed no indication of differences in PocR expression or stability (Figure 8C).

**Figure 8:**
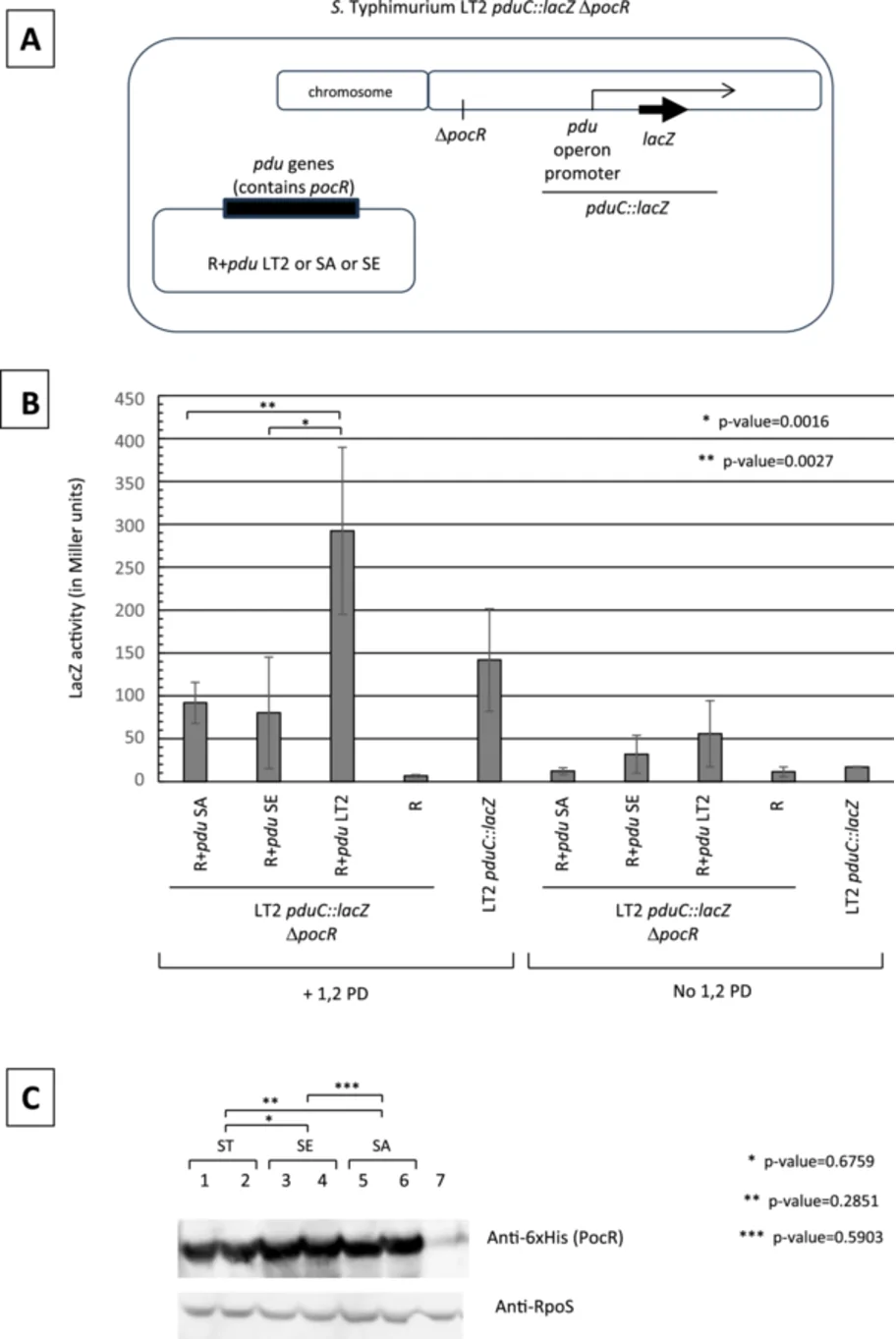
(A) Diagram of *S*. Typhimurium *pduC::lacZ* Δ*pocR* strain to test PocR activation of *pdu* operon using different *pocR* genes. The *lacZ* gene was transcriptionally fused to the chromosomal *pduC* gene (the third gene of the *pdu* operon), and the chromosomal *pocR* gene was deleted (Δ*pocR*). The plasmids R + *pdu* LT2, R + *pdu* SA, R + *pdu* SE (or plasmid vector) were individually transferred to this strain as the source of *pocR* for testing. Note that the diagram is highly schematic and not drawn to scale. (B) Results of LacZ assays using the indicated strains. The *p* values were obtained via *t*‐tests of the indicated sample comparisons using eight independent samples for each strain. (C) Western blot using total cell lysates of the reporter strain background containing plasmids pBAD18 + *pocR* ST nat, pBAD18 + *pocR* SE nat, or pBAD18 + *pocR* SA nat (noted as ST, SE, or SA, respectively). Samples were probed with anti‐6xHis antibodies (the *pocR* alleles are 6xHis‐tagged) and anti‐RpoS antibodies (serving as a normalization control). ImageJ analysis was used to quantify signals using the PocR (anti‐6xHis) signal normalized to the anti‐RpoS signal, and p‐values of the indicated comparisons were calculated via *t*‐tests using 6 independent samples for each.

## 4 Discussion

Overall, this work expands our ability to clone, transfer, and express *pdu* regions from different genomes, and it uncovered a previously unrecognized greater dependence on the Crp regulator for *S*. Arizonae and *S*. Enteritidis *pdu* expression compared to *S*. Typhimurium. This dependence could be alleviated by overexpressing the PocR protein from a coresident plasmid, indicating that greater PocR amounts can substitute for Crp at the *pdu* operon promoter. This could have important implications when using certain cloned *pdu* regions in possible Crp‐defective bacterial backgrounds. We present two different *pocR* alleles for our inducible PocR system (*pocR* opt and *pocR* sub), which gives greater flexibility in possible downstream applications depending on the background, cloned pdu region, etc. For example, the *pocR* sub allele provides a “non‐leaky” option for situations where the *pdu* system needs to be fully off when not being utilized, and the *pocR* opt allele provides the ability for greater PocR expression, which may be desired in certain situations.

Another significant outcome from this work was to overcome roadblocks to cloned *pdu* expression as seen in the more distantly related species *P. putida* and *P. carotovorum*. Our work indicates two different types of roadblocks at the transcriptional level. In *P. putida*, the roadblock can be overcome by overexpressing PocR, which indicates that, as expressed from the cloned *pdu* system *pocR* gene, PocR is less functional for activation and/or is not expressed/stable enough to achieve proper *pdu* operon activation. However, the *pdu* promoter/PocR system is still functional overall in this species. In *P. carotovorum*, PocR overexpression did not work, and we needed to replace the *pdu* operon promoter with a heterologous promoter. This indicates that in this species, the *pdu* promoter/PocR system does not function under the conditions used here. However, in both species, once the transcriptional roadblock is overcome, the necessary Pdu proteins are expressed and functional for 1,2‐PD metabolism (i.e., there appears to be no posttranscriptional barrier at the protein level). We do not know the exact mechanism of the roadblocks in *P. putida* and *P. carotovorum*. It could be that, in these species, PocR has reduced or lost DNA binding at the *pdu* promoter, or PocR has reduced or abrogated ability to activate transcription once bound, or PocR protein stability is reduced/absent, which results in lower overall PocR levels. However, we can observe PocR protein expression from pJAK12 + *pocR* opt in these species, as seen via the 1,2‐PD phenotype and via Western blot. Further studies will be needed to determine the specific nature of these roadblocks, but regardless, the PocR overexpression system and the use of a heterologous promoter provide viable options to potentially overcome barriers to cloned *pdu* expression in nonpermissive species.

To our knowledge, the data here are the first to suggest the concept that PocR proteins from different *Salmonella* serovars possess different abilities to activate *pdu* operon expression, as seen in both Figure 5 (Crp result) and Figure 8 (LacZ result). We do not know the specific reasons for this observation, but differences in PocR protein action and/or PocR expression/stability could contribute. There are amino acid differences between the different serovar PocR proteins, including in the HTH DNA‐binding region, though overall conservation is high (Figure A4). In addition, there are 81 SNPs on the entire *pduF‐pocR* transcript that could possibly differentially affect *pocR* RNA stability and/or translation (which could in turn affect PocR ability to activate the *pdu* operon). We tested the 233‐bp (ST, SE)/195‐bp (SA) sequence upstream of the *pocR* start codon when comparing PocR translation via Western blot (Figure 8C), and we observed no evidence of a difference. Thus, it is possible that other sequence differences on the larger native *pduF‐pocR* RNA could affect RNA stability/translation and subsequent *pdu* operon activation by PocR. Targeted mutation/swapping of any amino acid or nucleotide differences would be a logical next step for subsequent studies. It is also possible that a co‐factor needed for greater Arizonae and Enteritidis PocR activity is missing in the *S*. Typhimurium background.

Differences in PocR activity and resulting *pdu* operon phenotypes can have possible implications when cloning/transferring *pdu* regions from different sources. The recipient background is also an important variable, as shown here (via Δ*crp*, *P. putida*, and *P. carotovorum* results). Cloned *pdu* regions could be used to engineer novel metabolic pathways in a recipient (either using native Pdu enzymes or other engineered enzymes) or used in applications to package proteins or other molecules for relatively straightforward purification and downstream outcomes such as drug/vaccine delivery, protein/molecule storage, chemical/nutritional supplementation, etc. The ultimate goal would be to conveniently utilize cloned *pdu* regions (or other gene systems) across bacterial backgrounds using strategies that can establish/modulate expression regardless of barriers that exist upon transfer to other species. The concepts shown here could also be used generally when cloning/transfering other MCP gene systems in a range of bacteria.

## Author Contributions

**Emma Kopp:** conceptualization (supporting), data acquisition and analysis (lead), draft writing (supporting). **Anh Nguyen:** conceptualization (supporting), data acquisition and analysis (lead), draft writing (supporting). **Ryan Allen:** conceptualization (supporting), data acquisition and analysis (lead), draft writing (supporting). **Daniel Edelman:** conceptualization (supporting), data acquisition and analysis (lead), draft writing (supporting). **Timothy Hunter:** conceptualization (supporting), data acquisition and analysis (lead), draft writing (supporting). **Anna Wilson:** conceptualization (supporting), data acquisition and analysis (lead), draft writing (supporting). **James W. Wilson:** conceptualization (lead), data acquisition and analysis (lead), draft writing (lead).

## Funding

The authors have nothing to report.

## Ethics Statement

The authors have nothing to report.

## Conflicts of Interest

None declared.

## Figures and Tables

**Figure A1:**
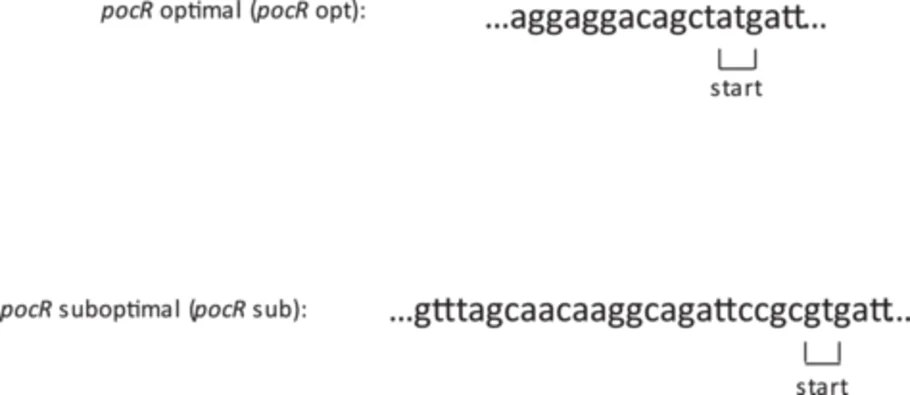
The translation signals used for the cloned *pocR* alleles of *pocR* opt and *pocR* sub.

**Figure A2:**
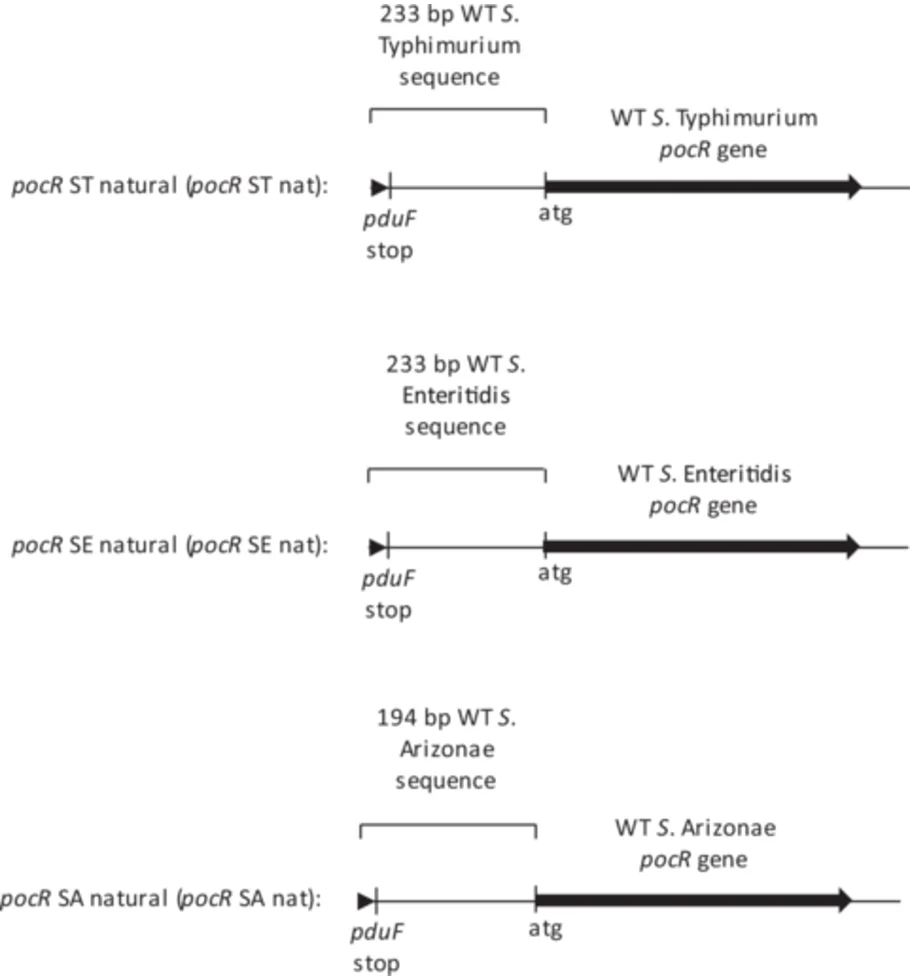
The sizes of native sequence used between *pduF* and *pocR* containing the natural translational signals for the corresponding *pocR* nat genes from *S*. Typhimurium, *S*. Enteritidis, and *S*. Arizonae.

**Figure A3:**
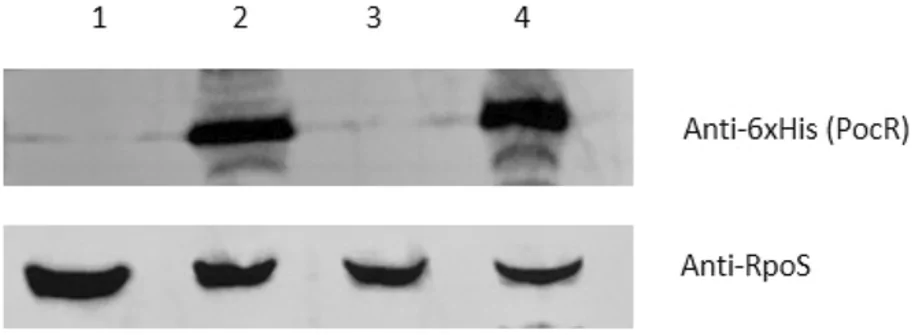
Western blot of total cell lysates of the *Pectobacterium carotovorum* strains indicated below, grown in LB medium to stationary phase with IPTG. Samples were probed with antibodies against 6xHis (for PocR opt) and against RpoS (for control). Samples are as follows: 1 = *P. carotovorum* (pJAK12); 2 = *P. carotovorum* (pJAK12 + *pocR* opt); 3 = *P. carotovorum* (R + *pdu* LT2, pJAK12); and 4 = *P. carotovorum* (R + *pdu* LT2, pJAK12 + *pocR* opt).

**Figure A4:**
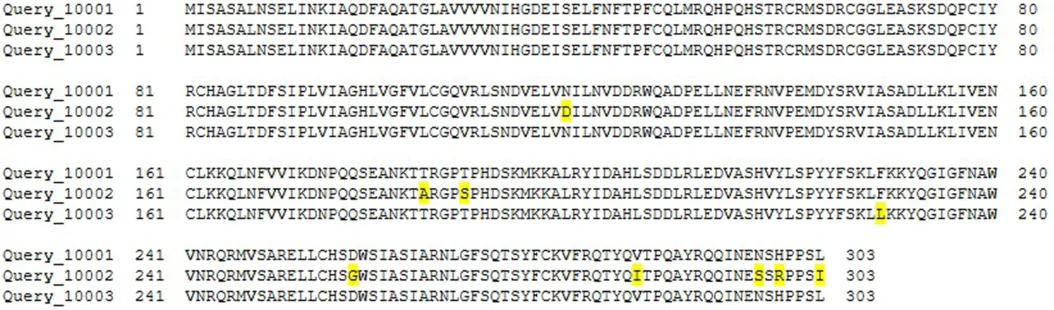
Alignment of the *S*. Typhimurium, *S*. Arizonae, and *S*. Enteritidis PocR protein sequences (accession numbers NC_003197, CBCSDD010000030, and CP195823 respectively). Amino acids in yellow are those that diverge. In the alignment, query_10001, query_10002, and query_10003 are Typhimurium, Arizonae, and Enteritidis, respectively.

## Data Availability

All data generated or analyzed during this study are included in this published article.

## 1

Figures A1, A2, A3, A4.
